# Cytologic and histologic cervical lesions and oncogenic HPV in women living with HIV (WLHIV) despite viral suppression: first evidence from Algeria

**DOI:** 10.1186/s13027-025-00717-4

**Published:** 2025-11-28

**Authors:** Mounira Rais, Amel Ouyahia, Meriem Abdoun, Sonia Taleb, Meriem Guechi, Noudjoud Amoura

**Affiliations:** 1 Department of Infectious Diseases, Faculty of Medicine, University Ferhat Abbas 1, University Hospital Center (CHU) of Sétif, Sétif, Algeria; 2 Department of Preventive Medicine and Epidemiology, Faculty of Medicine, University Ferhat Abbas 1, Sétif, Algeria

**Keywords:** HIV, Human papillomavirus (HPV), High-Risk HPV genotypes, Cervical intraepithelial neoplasia (CIN), Pap smear (Papanicolaou test), Colposcopy, Women living with HIV (WLHIV)

## Abstract

**Background:**

Women Living with HIV (WLHIV) are at increased risk of persistent high-risk Human Papillomavirus (HR-HPV) infections and cervical cancer. However, data on HPV genotype distribution and cervical cytologic abnormalities remain limited in North Africa, particularly among women receiving effective antiretroviral therapy (ART).

**Objective:**

To assess the frequency of cytologic abnormalities in WLHIV in Sétif, Algeria, identify HR-HPV genotypes, and evaluate associated immunovirological factors.

**Methods:**

A cross-sectional study was conducted from January to December 2024 at the HIV/STI/AIDS Reference Center in Sétif. WLHIV aged ≥ 18 years who provided informed consent were included. Each participant underwent a gynecological examination, cervical cytology (Pap smear), HR-HPV genotyping by molecular biology, and immunovirological assessment (CD4 T-cell count, HIV viral load). Colposcopy and biopsy were performed when indicated.

**Results:**

Among 115 enrolled participants, 100 smears were interpretable. Cytologic abnormalities : Atypical Squamous Cells of Undetermined Significance (ASC-US), Low-Grade/High-Grade Squamous Intraepithelial Lesions LSIL, HSIL) were found in 28% of WLHIV. HR-HPV infection was detected in 32% of participants, mainly genotypes HPV52, HPV16, and HPV18. A significant association was observed between HR-HPV positivity and high-grade lesions (HSIL) (*p* = 0.018; OR = 9.57, 95% CI: 1.02–89.48).

**Conclusion:**

WLHIV in Algeria show a high prevalence of HPV-related cytologic abnormalities, even with adequate immune status and viral suppression. These findings emphasize the importance of regular cervical screening and support current global recommendations for comprehensive prevention strategies, including HPV testing and vaccination.

## Introduction

High-risk human papillomavirus (HPV) genotypes are the main cause of cervical cancer, and persistent infection with these viruses increases the risk of developing precancerous lesions. Women living with human immunodeficiency virus (WLHIV) have a significantly increased risk of cervical intraepithelial neoplasia (CIN) compared with the general population [1]. This higher susceptibility is related to a greater prevalence of oncogenic HPV genotypes among WLHIV [2, 3]. Additionally, cervical intraepithelial lesions, which are often fragile and highly vascularized, may contribute to HIV transmission or reinfection with new viral variants [4].

In this patient population, infection with high-risk HPV genotypes is associated with a higher incidence of high-grade squamous intraepithelial lesions (HSIL), and the severity of these lesions correlates with immunosuppression, particularly low CD4 counts [5, 6], as well as the presence of certain oncogenic genotypes such as HPV16, 18, 31, 52, and 58 [7–9]. Previous studies have reported that precancerous cervical lesions are approximately 2.4 times more frequent in HIV-positive women than in the general population [10, 11]. Without adequate treatment, these lesions may persist in 60–90% of HIV-positive women compared with only about 20% in HIV-negative women [12–15].

To date, no study has been published in Algeria on cervical smears (CS) in WLHIV, representing a significant gap in national data. Therefore, the aim of this study is to estimate the prevalence of cervical cytological abnormalities, characterize the detected high-risk HPV (HPVHR) genotypes, and analyze the associated clinical, immunological, and virological factors in WLHIV monitored at the HIV/STI/AIDS reference center in Sétif.

## Materials and methods

### Study design and setting

This was a cross-sectional study conducted at the HIV/STI/AIDS Reference Center in Sétif, Algeria, from January to December 2024. Patients were recruited daily during routine consultations.

### Study population

The study included all HIV-positive women aged ≥ 18 years attending the center for follow-up or initial assessment who provided verbal informed consent were included. Pregnant women or those with prior hysterectomy were excluded.

### Data collection

Data were collected through structured interviews and clinical records, categorized into:

- Sociodemographic variables: age, marital status, education, occupation.
- Behavioral and reproductive history: age at first intercourse, parity, contraceptive use.
- Clinical and laboratory variables: ART regimen, CD4 T-cell count, HIV viral load, and HR-HPV results.

The standardized questionnaire used for data collection is attached as Supplementary Material 1.

### Clinical and laboratory procedures

#### Cervical cytology

Samples were collected using Ayre’s spatula and cytobrush, fixed in 95% ethanol, and stained using the Papanicolaou method. Cytological interpretation followed the 2001 Bethesda System by two independent cytopathologists; discrepancies were resolved by consensus.

#### DNA isolation and HPV genotyping

HPV genotyping was performed with the Linear Array HPV Genotyping Test (Roche Molecular Systems, Inc., New Jersey, and USA) according to the manufacturer’s instructions. This method allows the detection of 37 different types of HPV.

#### Blood sample for CD4 count and plasma HIV viral load

Women with abnormal smears were referred for colposcopy, interpreted using the French Society of Colposcopy grading system (TAG1: grade 1 atypical transformation, TAG2: grade 2 atypical transformation). Histological classification (CIN1, CIN2, CIN3, microinvasive/invasive cancer)., Targeted cervical punch biopsies were performed for suspected precancerous or cancerous lesions. Biopsies were processed according to WHO independently reviewed by two pathologists; discrepancies were resolved by consensus.

### Statistical analysis

The primary outcome was presence and grade of cervical cytological abnormality.

The main predictor was HR-HPV infection status. Continuous variables were summarized as medians (IQR); categorical variables as frequencies (%).

Associations were analyzed using Chi-square or Fisher’s exact test. Odds ratios (ORs) with 95% confidence intervals (CIs) were calculated. A p-value < 0.05 was considered statistically significant. Analyses were performed using SPSS version 26.0 (IBM Corp., Armonk, NY, USA).

### Ethical considerations

The study adhered to the Declaration of Helsinki and was approved by the Ethics Committee of the University Hospital Center of Sétif, Algeria. Due to sociocultural sensitivities related to HIV, the committee authorized verbal informed consent.

## Results

### Descriptive study

Of the 115 women included, 100 (87%) had an interpretable cervical smear (CS). The mean age was 43.6 years (range: 24–76), with the 31–45 age group predominating (52%). Most participants were married (53%) and housewives (88%). Educational attainment was: no schooling 26%, primary 31%, secondary 32%, university 11%. The mean age at first sexual intercourse was 22.8 years; 77% of participants had an HIV-positive partner who is also followed up at our STI/HIV/AIDS Reference Center. The mean number of pregnancies was 3.6, with 54% multiparous.

**The overall sociodemographic and gynecological characteristics of the study population are summarized in** Table 1.

**Table 1:** Sociodemographic and gynecological characteristics of the patients (*n* = 100)

Characteristics	*n* (%) / Mean ± SD
Age (years)	Mean = 43.6 ± 12.4 (range 24–76); majority aged 31–45 years (52.0%)
< 30	8 (8.0)
30–39	27 (27.0)
40–49	36 (36.0)
≥ 50	29 (29.0)
Marital status	
Married	53 (53.0)
Single	2 (2.0)
Widowed	31 (31.0)
Divorced	14 (14.0)
Educational level	
None	26 (26.0)
Primary	31 (31.0)
Secondary	32 (32.0)
University	11 (11.0)
Occupation	
Housewife	88 (88.0)
Employed / Self-employed	12 (12.0)
Place of residence	
Urban	79 (79.0)
Rural	21 (21.0)
Age at first sexual intercourse (years)	Mean = 22.8 ± 5.7;
< 15	5 (5.0)
15–20	37 (37.0)
>20	58 (58.0)
Parity	
Nulliparous	46 (46.0)
Multiparous	54 (54.0)
Contraceptive use	
None	73 (73.0)
Hormonal methods	15 (15.0)
IUD / Barrier	12 (12.0)
History of genital infections	32 (32.0)
Menstrual status	
Regular	61 (61.0)
Irregular	19 (19.0)
Menopausal	20 (20.0)
Previous Pap smear	
Yes	21 (21.0)
No	79 (79.0)
HIV-positive partner (followed at STI/HIV/AIDS Reference Center)	77 (77.0)

### Immuno-virological status

Immunovirrological status: all patients infected with HIV-1 and on ART. Mean duration since HIV diagnosis 4.8 years (range 6 months–19 years; SD 4.44). CD4 distribution: 58% >500/mm³, 29% 200–500/mm³, 13% <200/mm³ (mean CD4 677.08/mm³; min 41, max 2227; SD 428.70). Mean viral load 2.93 log (min 1.60; max 6.27). Overall 45% had detectable viral load (Table 2) and 55% had undetectable viral load per laboratory threshold.

**Table 2:** Clinical, immunological, and virological characteristics of the study population (*n* = 100)

Characteristics	*n* (%) / Mean ± SD	Range / Category
HIV type	HIV-1: 100 (100%)	—
Patients on ART	100 (100%)	—
Duration since HIV diagnosis (years)	4.8 ± 4.44	0.5–19
CDC classification	A: 16 (16%) B: 4 (4%) C: 80 (80%)	—
CD4 T-cell count (cells/mm³)	677.1 ± 428.7	41–2227
<200	13 (13.0%)	Severe immunosuppression
200–500	29 (29.0%)	Moderate immunosuppression
>500	58 (58.0%)	Preserved immunity
HIV viral load (log₁₀ copies/mL)	Mean = 2.93	1.60–6.27
Undetectable (< 1.60 log₁₀)	55 (55.0%)	Below detection limit
Detectable (≥ 1.60 log₁₀)	45 (45.0%)	Detectable viral replication

*Detection limit of the assay: 1.60 log10 copies/mL

“A viral load was considered detectable when ≥ 1.60 log10 copies/mL (corresponding to 40 copies/mL, the detection threshold of the assay used)”

**Detailed clinical**,** immunological**,** and virological characteristics are presented in** Table 2.

### Cervical cytology

Among 100 interpretable smears, 66 (66%) were abnormal, significantly more frequent in HPVHR + women (81.3%; *p* = 0.02; OR: 0.33; 95% CI: 0.12–0.90) (Table 3). Inflammatory smears were observed in 56%, LSIL in 13%, HSIL in 5%, and ASC-US in 10%. HR-HPV predominated in abnormal smears, notably HPV52, 16, 18, 31, and 58. Viral typing revealed single and multiple infections;

**Table 3:** Distribution of high-risk HPV genotypes and associated cytological findings (*n* = 32 HPVHR-positive samples)

HPV Genotype	Prevalence among HPVHR-positive samples (%)	Associated Cytological Findings (Bethesda classification)
HPV52	25.0	Inflammatory (12), LSIL (3), Normal (1)
HPV16	15.6	HSIL (2), LSIL (2), ASC-US (1)
HPV18	15.6	Inflammatory (4), LSIL (1), Normal (1)
HPV58	15.6	LSIL (2), ASC-US (1), Inflammatory (2)
HPV31	12.5	HSIL (1), LSIL (1), Inflammatory (2)
HPV56	12.5	Inflammatory (3)
HPV35	9.4	LSIL (1), Inflammatory (2)
HPV51	9.4	Inflammatory (3)
HPV59	9.4	ASC-US (1), Inflammatory (2)
HPV66	9.4	ASC-US (1), LSIL (1), Inflammatory (1)

Abbreviations: LSIL – Low-grade squamous intraepithelial lesion; HSIL – High-grade squamous intraepithelial lesion; ASC-US – Atypical squamous cells of undetermined significance

The distribution of cytological abnormalities and their associations with HR-HPV infection are shown in Table 3, while the statistical associations are detailed in Table 4.

**Table 4:** Association between high-risk HPV infection and cytological findings (*n* = 100)

Cytological Abnormality	Total *n* (%)	HR-HPV-positive *n* (%)	*p*-value	Odds Ratio (95% CI)
Inflammatory smear	56 (56.0)	24 (75.0)	0.009 *	3.37 (1.33–8.56)
LSIL	13 (13.0)	4 (12.5)	0.91	0.93 (0.26–3.30)
HSIL	5 (5.0)	4 (80.0)	0.018 *	9.57 (1.02–89.48)
ASC-US	10 (10.0)	4 (40.0)	0.56	1.47 (0.38–5.64)
Normal smear	34 (34.0)	6 (17.6)	—	—

*Statistically significant at *p* < 0.05

Abbreviations: HR-HPV – High-risk human papillomavirus; LSIL – Low-grade squamous intraepithelial lesion; HSIL – High-grade squamous intraepithelial lesion; ASC-US – Atypical squamous cells of undetermined significance

### HPV prevalence and genotypes

Overall HPV prevalence was 32%, all high-risk genotypes (HPVHR). Among HPVHR + women, 25% had co-infection with low-risk HPV (HPVBR). Most frequent genotypes: HPV52 (25%), HPV16/18/58 (15.6%), HPV31/56 (12.5%), HPV35/51/59/66 (9.4%). **The prevalence of individual HPV genotypes and their associated cytological findings are summarized in** Table 3.

### Colposcopy and histology 

Colposcopy was performed in 31 patients (40.6% HPVHR+). Colposcopic abnormalities were observed in 64.5%: TAG1 (40%) and TAG2 (60%). Histology identified 19 chronic cervicitis, 2 CIN1, 3 CIN2, 1 CIN3, and 1 carcinoma in situ. The carcinoma in situ patient underwent total hysterectomy with adnexectomy. Histology confirmed all high-grade lesions, and discrepancies were resolved by consensus review.

## Discussion

In this study, cytological abnormalities were frequent among WLHIV, with inflammatory lesions representing the majority (56%). This predominance aligns with the findings of Koffi et al. in Bangui, who also reported a high proportion of inflammatory lesions in WLHIV [10]. Such lesions likely reflect local mucosal immune dysregulation, which increases susceptibility to opportunistic infections commonly observed in immunocompromised patients.

Cervical dysplastic lesions were observed in 28% of women, including LSIL, HSIL, and ASC-US. This prevalence is much higher than in the general Cameroonian population (3.9%) [16] and exceeds the 5.01% prevalence reported among WLHIV in the Central Region of Cameroon. These results reinforce the established role of HIV as a major risk factor for the persistence of HPV infection and progression to cervical dysplasia.

Nevertheless, our prevalence remains lower than that reported by Atashili et al. (43.5%) among WLHIV initiating ART in Cameroon [17] and by Firnhaber et al. in South Africa (49.8%) [18]. This difference may be partly explained by immunological status, as Atashili’s cohort had a median CD4 count of 179 cells/mm³ compared with 677.08 cells/mm³ in our study. The association between immunosuppression, persistent HPV infection, and the development of cervical lesions has been well documented. Conversely, lower prevalence rates were described by Anorlu et al. in Nigeria (10.9%) [19], highlighting the influence of local epidemiological and immunological factors on HPV-related disease burden.

In terms of lesion type, we observed a predominance of low-grade squamous intraepithelial lesions (LSIL) (13%), while high-grade lesions (HSIL) accounted for 5%. This pattern differs from the results of Mogtomo Martin Luther Koanga et al. in Douala, who found a majority of HSIL (55.7%) in WLHIV [20]. These variations may be linked to differences in population characteristics, duration of HIV infection, ART status, and HPV genotype distribution. The significant association between HR-HPV infection and HSIL in our population (*p* = 0.018) confirms the oncogenic potential of these genotypes even among patients with preserved immunity, as also shown in the studies of Massad et al. and Clifford et al. [5, 6].

The most frequent HPV genotypes identified were HPV52, HPV16, and HPV18. This genotype profile resembles the distribution reported in sub-Saharan Africa [21, 22] but differs from that of European populations, where HPV16 predominates almost exclusively [15]. Regional variations in HPV genotype prevalence may reflect differences in sexual behavior, immune status, and viral ecology. The high proportion of HPV52 found in our study suggests that future preventive strategies in Algeria should consider this genotype’s epidemiological weight when implementing molecular screening tools.

Although the majority of our participants had satisfactory immunological status (median CD4 = 645 cells/mm³) and viral suppression (55% undetectable load), cervical abnormalities remained frequent. This finding echoes the observations of Clifford et al. [6] and Massad et al. [7], who reported that persistent HR-HPV infection and cervical dysplasia can occur even among women with controlled viremia. These data underline the need for continued gynecological surveillance in WLHIV regardless of ART success.

Regionally, Ouladlahsen et al. (2018) in Morocco reported an HPV prevalence of 74.5% among WLHIV, with 75% carrying high-risk genotypes and 13.8% presenting abnormal cytology [21]. Similarly, Belglaiaa et al. in the Souss Massa region found 39.3% high-risk HPV and 18% cytological abnormalities, with HIV infection increasing the risk almost fourfold (OR ≈ 3.98) [22]. These regional data confirm that the burden of HR-HPV infection among WLHIV is substantial across North Africa, although absolute prevalence varies by population and screening method.

Beyond Africa, data from the Middle East also underscore the importance of HPV surveillance. Mroueh et al. in Lebanon found an HPV prevalence of 4.9% in the general population, with HPV16 carriers exhibiting more abnormal smears (6.6% vs. 1.6%, *p* < 0.05) [23]. Although not specific to HIV infection, this observation emphasizes the oncogenic potential of persistent high-risk HPV even in populations with low HIV prevalence.

Our results, showing that HR-HPV infection was significantly associated with HSIL but not with lower-grade lesions, suggest that immune reconstitution under ART may limit the progression of early lesions but does not entirely prevent the emergence of high-grade neoplasia. This observation has also been documented in longitudinal cohorts [8, 9, 11]. Therefore, the persistence of HR-HPV infection despite immune recovery highlights the importance of ongoing screening rather than reliance solely on immunological markers.

### Study limitations

This study has certain limitations. Its cross-sectional design precludes establishing causality between immunological parameters and cervical lesion development. The sample size was relatively small and limited to one reference center, which may reduce generalizability. Moreover, histological confirmation was not systematically performed in all patients with abnormal cytology. Finally, behavioral factors such as condom use and smoking were self-reported, potentially introducing recall bias.

### Policy and research implications

Our findings underscore the need to integrate routine cervical cytology and HPV testing into HIV care programs. Strengthening such integrated services could improve early detection and reduce progression to invasive cervical cancer among WLHIV. In the Algerian context, molecular screening protocols should include genotypes most frequently detected here (HPV52, 16, 18). Future research should include longitudinal studies with larger multicentric samples to explore lesion persistence, genotype dynamics, and the long-term impact of ART duration on cervical pathology.

## Conclusion

In WLHIV attending a reference center in Sétif, Algeria, cervical cytological abnormalities and HPVHR infection are common. HPV52 and HPV16 were the most frequent genotypes, and HPVHR infection was associated with abnormal and high-grade cytology. These results support systematic cytological screening and HPVHR typing in WLHIV and structured colposcopic follow-up for abnormal findings. Future multicenter, longitudinal studies with control groups are needed to inform national screening recommendations.

## Data Availability

No datasets were generated or analysed during the current study.
